# The impact of endogenous estrogen exposures on the characteristics and outcomes of estrogen receptor positive, early breast cancer

**DOI:** 10.1007/s12672-021-00420-x

**Published:** 2021-08-17

**Authors:** Yasmin Korzets, Orly Yariv, Raz Mutai, Assaf Moore, Tzippy Shochat, Rinat Yerushalmi, Hadar Goldvaser

**Affiliations:** 1 Institute of Oncology, Tel Aviv Sourasky Medical Center, Weizmann St 6, Tel Aviv, Israel; 2 Sackler Faculty of Medicine, Tel Aviv University, Chaim Levanon St 30, Tel Aviv, Israel; 3 Institute of Oncology, Rabin Medical Center, Zeev Jabutinsky Rd 39, Petah Tikva, Israel; 4 Statistical Consulting Unit, Rabin Medical Center, Zeev Jabutinsky Rd 39, Petah Tikva, Israel; 5 Shaare Zedek Medical Center, The Oncology Institute, 12 Shmuel Bait St., PO Box 3235, 9103102 Jerusalem, Israel; 6 The Faculty of Medicine, The Hebrew University, Ein Kerem, P.O. Box 12271, 9112102 Jerusalem, Israel

**Keywords:** Breast cancer, Estrogen, Parity, Menopause, Menarche

## Abstract

**Background:**

Menstrual and parity history might impact the risk for breast cancer. Data on the impact of these factors on other tumor characteristics are limited.

**Methods:**

A single center retrospective cohort study comprising all women with estrogen receptor (ER) positive, human epidermal growth factor receptor 2 (HER2) negative, early breast cancer whose tumors were sent to OncotypeDX analysis. The prespecified subgroups were investigated: age of menarche (< 12 vs. ≥ 12 years), number of deliveries (0 vs. ≥ 1 childbirth and ≥ 5 childbirth vs. other), age of first delivery (≥ 30 years vs. younger age) and postmenopausal compared to premenopausal. The impact of age of menopause was also assessed categorically, using early (< 45 years) and late age of menopause (> 55 years). Differences in tumor characteristics were evaluated using T-test or Mann Whitney for continuous variables or Fisher’s exact test for categorical variables. Outcomes were assessed by Kaplan–Meier survival analysis, with the log-rank test.

**Results:**

A total of 620 women were included. After median follow-up of 10.4 years, early menopause was associated with significantly worse disease-free survival (HR = 2.26, p = 0.004) and overall-survival (HR = 2.60, p = 0.004), and multiparity was associated with significant worse disease-free survival (HR = 2.16, p = 0.026). These differences remain significant in multivariate analyses. Post-menopausal women were more likely to have stronger ER intensity (p = 0.002) but progesterone receptor (PR) positivity was less frequent (p = 0.009(. Early age of menarche was associated with PR positivity (p = 0.039). No other associations were found between the evaluated subgroups and tumor characteristics.

**Conclusions:**

The impact of endogenous estrogen exposure had little effect on breast cancer characteristics of early stage, luminal disease. Early menopause and multiparity were associated with worse outcome.

**Supplementary Information:**

The online version contains supplementary material available at 10.1007/s12672-021-00420-x.

## Introduction

Breast cancer accounts for 30% of all cancer diagnosed in women, with a lifetime risk of 12.4% [1]. Estrogen and its metabolites have a role in breast cancer development. Exposure to high levels of either endogenous or exogenous estrogen increase the risk of breast cancer, particularly estrogen receptor (ER) positive breast cancer [2], which is overexpressed in approximately 75% of all breast cancers [3]. In contrast to exogenous estrogen exposure, which is determined by treatment women might take during their life time such as oral contraceptive or hormone replacement therapy, endogenous estrogen exposure is determined by events women are experiencing during their life time, including duration of ovulations (i.e. time between menarche and menopause) and pregnancies.

Excess risk of breast cancer has been attributed to early menarche and late menopause, this is associated to lengthening woman’s reproductive years [4]. Compared to early menarche, late menarche is associated with decreased risk for breast cancer which is more robust for ER positive disease than ER negative disease [5]. Late menopause independently increases the risk for breast cancer, with a relative increase of 3% for every year older at menopause [4].

Parity also effects breast cancer risk through hormonal mechanisms. Nulliparity is related to an increase risk of breast cancer, whereas multiparity has an overall protective effect, although there is an increased risk for breast cancer during the first two decades after childbirth and then it gradually decreases [6, 7]. Age at first full-term pregnancy is another factor related to breast cancer risk, with lower breast cancer risk in women with younger age of first birth [8]. Obesity is a known risk factor for cancer, higher BMI increases the risk for ER positive breast cancer specifically in postmenopausal women, possibly explained by higher estrogen levels resulting from the peripheral conversion of estrogen precursors from adipose tissue [9, 10].

The impact of endogenous estrogen on breast cancer characteristics is less known. Earlier age at menarche and older age of menopause are associated with more frequently lobular tumors [4]. Older age at menopause is associated with the development of ER positive tumors rather than to ER negative tumors [4]. An association between the baseline levels of sex-hormone has also been described in postmenopausal women, with higher level of estradiol and testosterone associated with increased risk of ER positive disease, but not with ER negative breast cancer [11, 12].

Breast cancer is a diversified disease, with different histopathological characteristics and molecular subtypes that determine both treatment and prognosis [13]. ER positive, human epidermal growth factor receptor 2 (HER2) negative-subtype represents approximately 65–70% of invasive breast cancer and compared to other subtypes have a better prognosis [13]. Yet, the luminal-subtype has a spectrum of variables affecting its prognosis and treatment, including the extent of the disease, grade, Ki67, intensity of ER and genomic risk [14–17]. One of the genomic assays used in this population is the 21-gene recurrence-score assay (Oncotype DX, genomic Health) which provides both predictive and prognostic information in early-stage luminal disease [18].

The aim of our study was to investigate the effect of endogenous estrogen on histological tumor characteristics and on the genomic risk in early breast cancer women with ER positive, HER2 negative disease and to explore the correlation of these exposures on outcomes.

## Methods

This was a retrospective single center cohort study. All women who were treated in our institute between 2005 and 2012 with ER positive, HER2 negative, early breast cancer whose tumors were sent to Oncotype DX analysis were included. As Oncotype DX was available and reimbursed in Israel since 2005, nearly all patients with luminal early breast cancer who were medically fit for adjuvant chemotherapy were sent for Oncotype DX. The Clalit Heath Services are the medical provider for the majority of the included patients in this cohort.

A detailed review of patients’ medical records and pathological reports was conducted. Data on demographics, adjuvant therapy and pre-specified clinical-pathological parameters were extracted including: tumor size (categorized as T ≤ 1 cm, 1 < T ≤ 2 cm and T > 2 cm), nodal status (negative or positive, including both macro- and micrometastases), intensity of ER and progesterone receptor (PR) expression, grade, Ki67, lymphovascular and perineural invasion and Oncotype DX recurrence score (RS). RS was categorized by the TailorX groups: low risk: RS ≤ 25 and high risk: RS > 25 [20]. ER/PR staining report was based on the modified version of H-score method [21] [(1× % cells + 1) + (2× % cells + 2) + (3× % cells + 3)]/100, yielding a score, ranges from 0 to 3. The intensity of hormone receptor staining was classified into 3 categories: weak expression level is defined as—0 < ER/PR ≤ 1, intermediate—1 < ER/PR ≤ 2 and strong—ER/PR > 2.

Data on endogenous estrogen exposure and parity were collected including: age of menarche, number of childbirth and age of first delivery, menopausal status and age of menopause. Data on any loco-regional, distant recurrence and death were also recorded. Data lock was in 18/06/2020.Time of recurrence was defined as the date of biopsy from site of recurrence or the date of abnormal imaging suggestive for metastatic disease. Disease-free survival (DFS) was defined as the time between breast surgery to an event (recurrence or death) or data lock. Duration of survival was defined as time from initial breast cancer diagnosis to date of death or time of data lock. Patients’ vital status was ascertained through Israel’s ministry of interior database.

The impact of endogenous estrogen exposure and parity on breast cancer characteristics and on outcomes was evaluated. Comparisons were conducted for the pre-specified subgroups including: age of menarche (age < 12 compared to age ≥ 12), nulliparity (yes compared to no), multiparity (women with 5 or more childbirths compared to less than 5 childbirths) and menopause (postmenopausal compared to premenopausal). For postmenopausal women comparisons were done for early menopause (age < 45 compared to menopause age ≥ 45) and late menopause (age > 55 compared to menopause at the age ≤ 55). This discrimination was applied based to prior data suggesting differences in breast cancer risks between these subgroups [22]. As data were analyzed anonymously, no consent was required. The study protocol was approved by our institutional ethics committee.

### Statistical analysis

Statistical analysis was generated using SAS Software, Version 9.4. Data were reported descriptively by each of the pre-specified categories as described above. Categorical variables were presented by as proportions and continuous variables were presented by mean and standard deviation (SD) or Median (Range) as appropriate. T-test for normally distributed variables or Mann Whitney for non normal were used to compare the value of continuous variables between study groups. Fisher’s exact test was used to compare the value of categorical variables between study groups.

The impact of age of menarche, menopause, age of menopause and parity on DFS and OS were assessed. Overall-survival (OS) and DFS were assessed by Kaplan–Meier survival analysis, with the log-rank test. For significant differences in outcomes in univariate analyses, multivariate analysis for age, tumor size, nodal status, grade and Oncotype RS were assessed by the Cox proportional hazards model. Two-sided p-values less than 0.05 were considered statistically significant.

## Results

We identified 705 patients with early breast cancer whose tumor were sent to Oncotype DX analysis at our institution from 2005 to 2012. 85 patients were excluded, remaining 620 women who were included in the study cohort.

Median age was 61 years (range 34–85 years), 75% (464) women were postmenopausal, most of them 86% menopaused before the age of 55 and 14% had menopause before the age of 45. Median age of menarche was 13 years and in 86% age of menarche was 12 and above. Median number of deliveries was 3 (range 0–14), 6% were multiparous and 10% were nullipara. Invasive ductal carcinoma (IDC) was the most common histology (81%), followed by invasive lobular carcinoma (ILC) (12%). Women were most likely to present with tumor size ≤ 2 cm (77%) and node negative disease (82%). Grade was well, moderately and poorly differentiated in 17%, 66% and 17% of tumors, respectively with strong ER intensity in 76% of women and negative PR in 14% of women. Ki67 was 20% or lower in 78% of tumors. Oncotype DX RS was 25 or lower in 82%. Perineural and angiolymphatic invasion were uncommon presenting in 9% and 6% of tumors, respectively. Overall, 77% of women with high genomic risk (RS > 25) were treated with adjuvant chemotherapy. 97.7% women were treated with adjuvant endocrine therapy. The initial endocrine therapy was tamoxifen for 87% and aromatase inhibitors for 10.7%. The administration of aromatase inhibitors was more common in postmenopausal women compared to pre-menopausal women (12.5% vs. 3.2%). In the other evaluated subgroups, the distribution of the initial endocrine therapy was similar, see Supplementary Table 1.

Histo-pathological characteristics by the pre-specified subgroups are presented in Table 1. Post-menopausal women were more likely to have stronger ER intensity (79% vs. 65%, p = 0.002), but PR negativity was significantly more frequent (16% vs. 7%, p = 0.009). Early age of menarche was associated with PR positivity (94% vs. 85%, p = 0.039). No other associations were found between endogenous estrogen exposures and tumor characteristics.

**Table 1:** The impact of endogenic estrogen exposure of tumor characteristics

N (%)	Age of menarche		Nulliparity		Multiparity (≥ 5 deliveries		Age at first delivery		Menopause		Early menopause (age < 45)		Late menopause (age > 55)	
	Yes (n = 17)	No (n = 401)	No (n = 353)	Yes (n = 65)	Yes (n = 464)	No (n = 131)	≥ 30 (n = 58)	< 30 (n = 284)	Yes (n = 36)	No (n = 569)	No (n = 543)	Yes (n = 62)	≥ 12 (n = 448)	< 12 (n = 69)
T size														
T ≤ 1 cm	12 (17%)	98 (22%)	16 (26%)	117 (21%)	128 (23%)	5 (14%)	55 (19%)	18 (31%)	27 (21%)	106 (23%)	15 (23%)	81 (23%)	92 (23%)	4 (23.5%)
1 < T ≤ 2	39 (57%)	244 (55%)	33 (53%)	297 (55%)	307 (54%)	23 (64%)	162 (57%)	32 (55%)	66 (50%)	259 (56%)	34 (52%)	198 (56%)	223 (56%)	9 (53%)
T > 2 cm	18 (26%)	105 (23%)	13 (21%)	128 (24%)	133 (23%)	8 (22%)	67 (24%)	8 (14%)	38 (29%)	98 (21%)	16 (25%)	73 (21%)	85 (21%)	4 (23.5%)
P value for the difference (T)	0.675		0.725		0.413		0.074		0.169		0.763		0.968	
Nodes														
Negative^a^	54 (79%)	374 (84%)	53 (85%)	448 (83%)	472 (83%)	29 (81%)	233 (83%)	48 (83%)	105 (80%)	386 (84%)	54 (84%)	293 (84%)	333 (84%)	14 (82%)
P value for the difference (N)	0.383		0.721		0.647		1.000		0.355		1.000		0.745	
Histology subtype^c^														
IDC	54 (79%)	360 (80%)	49 (79%)	440 (81%)	462 (81%)	27 (75%)	227 (80%)	45 (78%)	106 (81%)	373 (81%)	54 (83%)	281 (80%)	320 (80%)	15 (88%)
ILC	13 (19%)	53 (12%)	7 (11%)	68 (13%)	69 (12%)	6 (17%)	40 (14%)	11 (19%)	15 (11%)	60 (13%)	8 (12%)	46 (13%)	53 (13%)	1 (6%)
Other^b^	2 (3%)	35 (8%)	6 (10%)	34 (6%)	37 (7%)	3 (8%)	17 (6%)	2 (3%)	10 (8%)	30 (6%)	3 (5%)	26 (7%)	28 (7%)	1 (6%)
P value for the difference (histological subtype)	0.114		0.584		0.640		0.506		0.824		0.704		0.653	
Grade														
1	6 (11%)	65 (18%)	11 (21%)	74 (17%)	80 (17%)	5 (19%)	38 (16%)	9 (20%)	24 (22%)	59 (16%)	9 (17%)	45 (16%)	51 (16%)	3 (19%)
2	37 (67%)	243 (66%)	35 (67%)	291 (65%)	308 (65%)	18 (69%)	152 (66%)	30 (65%)	67 (61%)	254 (66%)	37 (68%)	188 (66%)	215 (66%)	10 (62%)
3	12 (22%)	61 (16%)	6 (12%)	82 (18%)	85 (18%)	3 (12%)	42 (18%)	7 (15%)	18 (17%)	67 (18%)	8 (15%)	53 (18%)	58 (18%)	3 (19%)
P value for the difference (grade)	0.510		0.581		0.331		0.900		0.444		0.899		0.562	
ER intensity^d^														
≤ 1	0	9 (2%)	2 (3%)	9 (2%)	10 (2%)	1 (3%)	6 (2%)	1 (2%)	5 (4%)	6 (2%)	0	5 (1%)	5 (1%)	0
1 < ER ≤ 2	17 (25%)	97 (22%)	18 (29%)	116 (21%)	126 (22%)	8 (22%)	64 (23%)	11 (19%)	41 (31%)	90 (19%)	10 (15%)	70 (20%)	79 (20%)	1 (6%)
ER > 2	52 (75%)	342 (76%)	42 (68%)	418 (77%)	433 (76%)	27 (75%)	214 (75%)	46 (79%)	85 (65%)	368 (79%)	55 (85%)	278 (79%)	317 (79%)	16 (94%)
P value for the difference (ER)	0.441		0.240		0.905		0.813		0.002		0.422		0.316	
PR														
Negative	4 (6%)	67 (15%)	6 (10%)	81 (15%)	83 (14%)	4 (11%)	41 (14%)	3 (5%)	9 (7%)	72 (16%)	10 (15%)	53 (15%)	61 (15%)	2 (12%)
P value for the difference (PR)	0.039		0.340		0.806		0.055		0.009		1.000		1.000	
PNI														
Yes	3 (4%)	21 (5%)	2 (3%)	23 (4%)	24 (4%)	1 (3%)	12 (4%)	0 (0%)	5 (4%)	22 (5%)	2 (3%)	18 (5%)	19 (5%)	1 (6%)
P value for the difference (PNI)	1.000		1.000		1.000		0.137		0.814		0.751		0.588	
LVI														
Yes	4 (6%)	27 (6%)	2 (3%)	31 (6%)	33 (6%)	0 (0%)	16 (6%)	3 (5%)	9 (7%)	25 (5%)	3 (5%)	21 (6%)	22 (6%)	2 (12%)
P value for the difference (LVI)	1.000		0.563		0.250		1.000		0.524		1.000		0.269	
Ki67														
> 20%	9 (17%)	75 (23%)	9 (18%)	88 (22%)	91 (22%)	6 (19%)	42 (21%)	13 (32%)	20 (23%)	75 (21%)	12 (24%)	57 (21%)	65 (22%)	4 (27%)
P value for the difference (ki67)	0.473		0.588		0.825		0.157		0.771		0.707		0.748	
Oncotype RS														
RS ≤ 25	56 (81%)	372 (83%)	54 (87%)	440 (81%)	461 (81%)	33 (92%)	236 (83%)	45 (78%)	106 (81%)	383 (83%)	54 (83%)	294 (83%)	333 (83%)	15 (88%)
RS > 25	13 (19%)	76 (17%)	8 (13%)	103 (19%)	108 (19%)	3 (8%)	48 (17%)	13 (22%)	25 (19%)	81 (17%)	11 (17%)	59 (17%)	68 (17%)	2 (12%)
P value for the difference (Oncotype)	0.732		0.300		0.124		0.347		0.698		1.000		0.749	

Data were not available for: age of menarche—n = 103, number of deliveries—n = 15, age of first delivery—n = 278, menopause—n = 25, age of menopause—n = 202

*ER* estrogen receptor, *IDC* invasive ductal carcinoma, *ILC* invasive lobular carcinoma, *OC* Oral Contraceptive, *PNI* perineural invasion, *PR* progesterone receptor, *LVI* lymphovascular invasion, *RS* recurrence score

^a^Node positive included micro-metastasis and marco-metastasis to lymph nodes

^b^Other subtypes include mixed ductal-lobular

^c^Other histology subtypes included medullary, mucinous, papillary and tubular carcinomas

^d^Intensity hormone receptor determined by the modified version of H-score method and was defined by the following categories: weak—0 < ER/PR ≤ 1, intermediate—1 < ER/PR ≤ 2, strong—ER/PR > 2

**Fig. 1:**
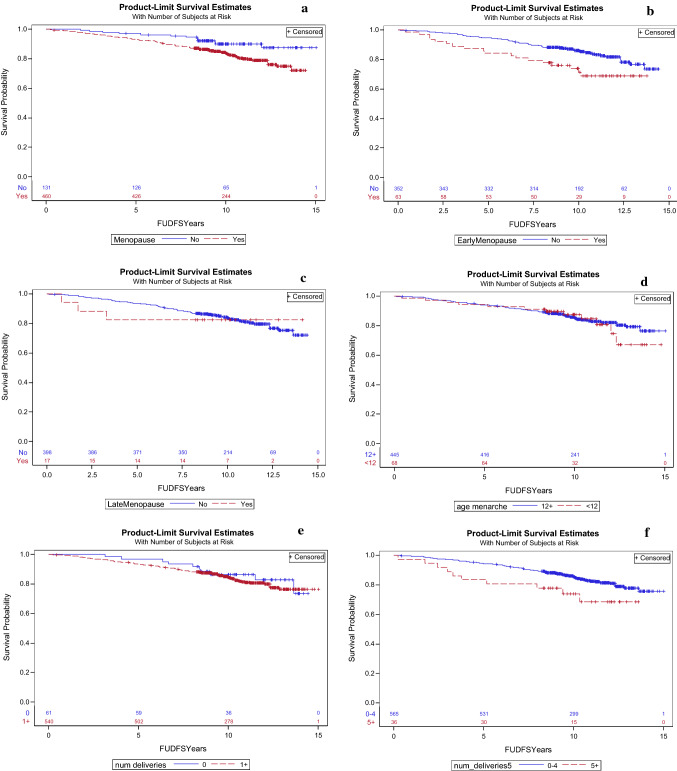

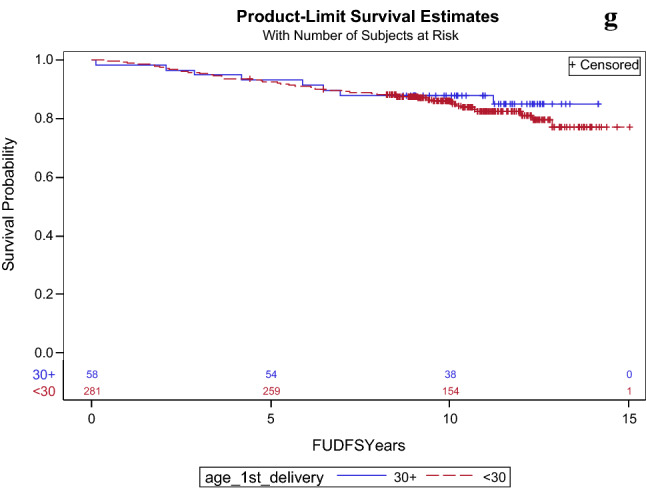
Disease free survival probabilities in **a** menopausal women, **b** early menopause (menopause before the age of 45), **c** late menopause (menopause older the age of 55), **d** early age at menarche (menarch before the age of 12), **e** nullipara, **f** number of deliveries (0–4 vs. 5 and more), **g** age at first live birth (30 years and older vs. < 30 years)

The median duration of follow-up was 10.4 years. The results of DFS are presented in Table 2 and Fig. 1. Estimated 10-year DFS was significantly worse for women with early menopause (71.3% vs. 86.3%), HR = 2.26, 95% CI 1.30–3.93, p = 0.004) and for multiparity (73.9% vs. 85.6%), HR = 2.16, 95% 1.10–4.25, p = 0.026. On multivariate analysis worse DFS remained to be significantly worse for women with early menopause and multiparous women (p = 0.004 and 0.01, respectively) together with oncotype RS (p < 0.001), see Table 3. Compared to premenopausal women, postmenopausal women had worse 10-year DFS that approached significance (83.8% vs. 90.1%, HR = 1.69, 95% 0.92–3.09, p = 0.09). This difference did not remain significant on multivariate analysis (p = 0.289). DFS was comparable in the other evaluated subgroups.

**Table 2:** DFS and OS and by estrogen exposure

	Estimated 10 year OS (95% CI)	OS—HR, 95% CI, p value	Estimated 10 year DFS (95% CI)	DFS—HR, 95% CI, p value
All	90.2% (87.4–92.4%)		85.7% (82.6–88.3%)	
Menarche Age < 12 (n = 69)	90.7%	1.11 (0.48–2.56) P = 0.815	87.4%	0.92 (0.44–1.88) P = 0.809
Menarche Age ≥ 12 (n = 448)	90.5%		85.4%	
Nulliparity—yes (n = 62)	89.6%	0.89 (0.39–2.04) P = 0.79	86.5%	1.09 (0.53–2.22) P = 0.818
Nulliparity—no (n = 543)	89.9%		84.7%	
Multiparity^a^—yes (n = 36)	84.9%	1.74 (0.72–4.23) P = 0.22	73.9%	2.16 (1.10–4.25) P = 0.026
Multiparity—no (n = 569)	90.2%		85.6%	
Age of first delivery < 30 (n = 284)	90.7%	1.18 (0.43–3.28) P = 0.748	86.0%	1.07 (0.49–2.37) P = 0.86
Age of first delivery ≥ 30 (n = 58)	92.7%		87.9%	
Menopause—yes (n = 464)	88.5%	2.62 (1.08–6.38) P = 0.034	83.8%	1.69 (0.92–3.09) P = 0.09
Menopause—no (n = 131)	95.8%		90.1%	
Early menopause^a^—yes (n = 65)	78.6%	2.60 (1.36–4.95) P = 0.004	71.3%	2.26 (1.30–3.93) P = 0.004
Early menopause—no (n = 353)	90.4%		86.1%	
Late menopause^a^—yes (n = 17)	94.1%	0.80 (0.15–4.15) P = 0.79	82.4%	1.48 (0.50–4.38) P = 0.478
Late menopause^a^—no (n = 401)	88.4%		83.9%	

Data on outcomes were not available: age of menarche—n = 103, number of deliveries—n = 15, age of first delivery—n = 278, menopause—n = 25, age of menopause—n = 202

*CI* confidence interval, *DFS* disease free survival, *HR* hazard ratio, *OS* overall survival

^a^Definitions: multiparity—five or more childbirths, early menopause—menopause before the age of 45, late menopause- menopause older the age of 55

**Table 3:** Multivariate analysis for OS and DFS

	Multivariate analysis OS				Multivariate analysis DFS			
	Menopausal status HR (95% CI)	P value	Early menopause HR (95% CI)	P value	Menopausal status HR (95% CI)	P value	Early menopause HR (95% CI)	P value
Menopause								
Yes vs. no	1.42 (0.45–4.48	0.553	–	–	1.57 (0.68–3.65)	0.289	–	–
Early menopause^a^								
Yes vs. no	–	–	2.54 (1.24–5.19)	0.011	–	–	2.46 (1.34–4.52)	0.004
Age	1.04 (1.00–1.08)	0.063	1.05 (1.00–1.10)	0.045	1.00 (0.97–1.04)	0.809	1.01 (0.97–1.05)	0.551
Tumor size								
T > 2 vs. T ≤ 2 cm	1.47 (0.76–2.86)	0.25	1.64 (0.78–3.42)	0.19	1.25 (0.71–2.20)	0.441	1.27 (0.66–2.44)	0.484
Nodal status^b^								
Positive vs. negative	1.55 (0.76–3.17)	0.233	1.13 (0.48–2.66)	0.776	1.33 (0.72–2.47)	0.366	1.27 (0.62–2.60	0.52
Grade								
Grade 2 vs. 1	1.45 (0.50–4.18)	0.493	1.09 (0.37–3.23)	0.872	1.22 (0.57–2.63)	0.609	1.01 (0.44–2.30)	0.99
Grade 3 vs. 1	1.04 (0.30–3.62)	0.946	0.83 (0.22–3.09)	0.781	0.88 (0.34–2.26)	0.791	0.89 (0.32–2.48)	0.825
Oncotype								
RS > 25 vs. RS ≤ 25	3.24 (1.70–6.18)	< 0.001	2.85 (1.37–5.96)	0.005	3.12 (1.83–5.31)	< 0.001	3.05 (1.64–5.67)	< 0.001
Multiparity^a^								
Yes vs. no	–	–	–	–	2.67 (1.20–5.99)	0.017	3.17 (1.31–7.66)	0.01

*CI* confidence interval, *DFS* disease free survival, *HR* hazard ratio, *OS* overall survival, *RS* recurrence score

^a^Definitions: early menopause—menopause before the age of 45, multiparity—five or more childbirths

^b^Node positive—include both micrometastases and macrometastases

**Fig. 2:**
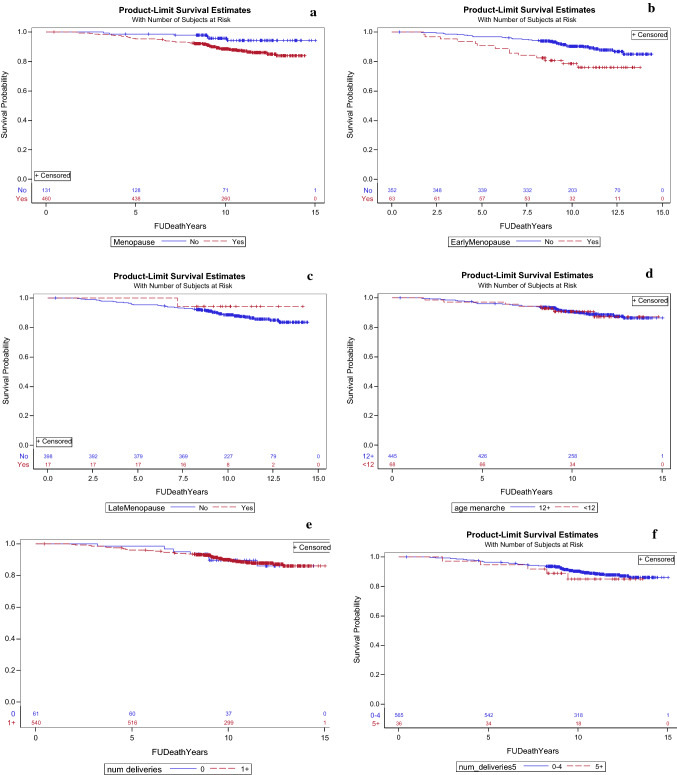

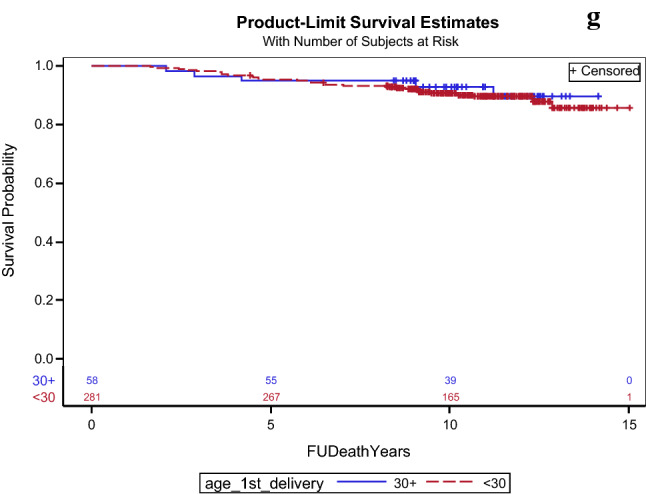
Overall survival probabilities in **a** menopausal women, **b** early menopause (menopause before the age of 45), **c** late menopause (menopause older the age of 55), **d** early age at menarche (menarche before the age of 12), **e** nullipara **f** number of deliveries (0–4 vs. 5 and more), **g** age at first live birth (30 years and older vs. < 30 years)

The results of OS are presented in Table 2 and Fig. 2. Women with early menopause had worse 10-year OS compared to women with menopause at later age (78.6% vs. 90.4%,), HR = 2.60, 95% CI 1.36–4.95, p = 0.004. In multivariate analysis the association between worse OS and early menopause remained significant (p = 0.011), together with older age (p = 0.011) and higher oncotype RS (p = 0.005). Compared to premenopausal women estimated 10-year overall survival was significantly worse in post-menopausal women (88.5% vs. 95.8%), HR = 2.62, 95% CI 1.08–6.38, p = 0.034. In multivariate analyses the menopausal status did not remain associated with worse OS (p = 0.553), Oncotype RS was the only variable that was associated with worse OS (p < 0.001), and the impact of older age on worse OS approached significance (p = 0.063). Results of the multivariate analyses are shown in Table 3. OS was comparable in the other evaluated subgroups. Of note, administration of chemotherapy for women in high genomic risk was significantly lower in post-menopausal women comparted to pre-menopausal women (70% vs. 96%, p = 0.006), but for early menopause and multipara women, rates of chemotherapy administration were comparable to their control.

## Discussion

Lifetime exposure to estrogen that is related to reproductive factors including early menarche, late menopause and parity have a well-established impact on breast cancer risk [2]. While the increased risk is more prominent for ER positive disease rather than ER negative disease, few studies address the effect of reproductive factors on other tumor characteristics and outcomes. Here, we investigated the influence of endogenous estrogen exposure on early-stage luminal breast cancer characteristics including also the impact on genomic risk. Overall, the impact of the evaluated endogenous exposures on breast cancer characteristics was relatively limited. Menopause was associated with stronger ER intensity, but with PR negativity. Women with early menarche were more likely to have PR positive disease. Oncotype RS, a well validated prognostic and predictive factor that is not a subject for inter-laboratory heterogeneity [19], was comparable in all of the evaluated subgroups. No other associations between parity, age of first delivery and age of menopause with tumor characteristics were identified.

Data suggest possible differential effect of reproductive risk factors and obesity according to breast cancer subtype, with parity, age at menarche, age at first birth, breastfeeding and obesity demonstrating stronger associations with luminal subtype compared to other subtypes [17, 20], however data on the effect of these factors of the intensity of ER and PR staining are scarce. In our analysis an association between early menarche and PR positivity was identified. This is in line with other studies showing a higher concordance of ER/PR positive breast cancer in women with early age of menarche [20, 23, 24].

In this study, we reported on the influence of endogenous estrogen exposures on outcome with a follow-up duration of more than 10 years. Interestingly, despite comparable tumor characteristics, early menopause and multiparity were each independently associated with worse outcomes, and these differences remained significant in multivariate analysis. Postmenopausal women also had worse OS compared to premenopausal women, but this difference did not remain significant in multivariate analysis and can probably be attributed to non-breast cancer mortality in older population.

Our finding of adverse outcomes of women who menopaused before the age of 45 is meaningful and might have important implication of treatment decisions. Menopause gives rise to cardiovascular morbidity and metabolic syndrome [25, 26]. Earlier age of menopause enhances the risk for cardiovascular morbidity and mortality [27–29]. Given the excellent prognosis of women with luminal early breast cancer, the most common cause of death in this population is cardiovascular disease [30, 31]. Adjuvant treatment for breast cancer might increase cardiovascular morbidity. Anthracycline based adjuvant chemotherapy that are often recommended to women with high genomic risk or to women with high clinical risk [18, 32], might cause cardiotoxicity. Adjuvant treatment with aromatase inhibitors, which are consider the standard of care in all postmenopausal women [33] and can often be considered for higher risk premenopausal in combination with ovarian function suppression [34], have increased odds for cardiovascular morbidity compared to both tamoxifen or placebo [35, 36]. Adjuvant radiation that is given to the vast majority of women after breast conserving surgery, is also associated with cardiovascular toxicity when treating left sided disease [37]. Tailoring treatment decisions based on the individual risk of breast cancer recurrence and on other comorbidities including risk of cardiovascular disease are required. Modifications to reduce treatment related cardiovascular toxicity, such as anthracyclines sparing chemotherapy [38], shorter treatment with AIs [35] and omission of radiation or partial breast irradiation [39, 40] should be considered. Our findings demonstrated that age of menopause might also be an important factor when weighing the risk and benefit balance of treatment options.

Multiparity was also associated with worse DFS. This finding is significant and is in line with previous publications [41, 42], however due to the low number of multiparous women, the conclusions that can be drawn are limited. Multiparity might be associated with low socioeconomic status [43, 44], and hence lower adherence to treatment and higher risk of comorbidities [45]. Multiparty is also an independent predictor for obesity [46, 47], which may also explain worse outcome after breast cancer diagnosis, both due inferior specific breast cancer survival [48] and non-breast cancer mortality [49]. These hypotheses might partially explain our finding, however, our data on weight and socioeconomic status were insufficient. Larger scales studies are needed to elucidate the effect on multiparity on breast cancer outcome.

We acknowledge the limitations of a single-center retrospective cohort with inherent biases. The histopathological variables were not re-evaluated for the study by central pathology. While data on adjuvant chemotherapy and on the initial adjuvant endocrine were collected, data on the duration of therapy, subsequent endocrine therapy (i.e., switch from tamoxifen to aromatase inhibitors) and the addition of ovarian function suppression for premenopausal were not available. Additionally, data on body mass index, an important variable that may affect breast cancer incidence and features [50], were incomplete and therefore were not addressed in this study. On the other hand, considering the scarcity of data on the effect of endogenous estrogen on breast cancer characteristics our cohort represents a large number of patients with long duration of follow up. The effect on all known prognostic pathological characteristics were meticulously collected and the assessment of endogenous estrogen impact on Oncotype DX score is novel.

In conclusion, our results suggest little impact of endogenous estrogen exposure on breast cancer characteristics of early stage, luminal disease. Early menopause and multiparity were associated with worse outcome. These findings might be related to other comorbidities or direct influence on breast cancer pathogenesis. Given the impact of early menopause and multiparty on outcome, these variables should be taken into consideration in treatment decisions on early-stage luminal breast cancer.

## Supplementary Information

Below is the link to the electronic supplementary material.

Supplementary file 1 (DOCX 17 KB)

## Data Availability

The datasets generated during and/or analysed during the current study are available from the corresponding author on reasonable request.
