# Supplementary material for: The impact of endogenous estrogen exposures on the characteristics and outcomes of estrogen receptor positive, early breast cancer
Source: Discov Oncol. 2021 Aug 17;12:26. doi: 10.1007/s12672-021-00420-x (PMC8777527; doi:10.1007/s12672-021-00420-x)
Supplement: Supplementary file 1 — (DOCX 17 KB) [file 12672_2021_420_MOESM1_ESM.docx]

**Supplementary table 1: Type of initial endocrine therapy by endogenic estrogen exposure**

| **P value** | **None, n (%)** | **Aromatase inhibitors, n (%)** | **Tamoxifen, n (%)** |  |
| --- | --- | --- | --- | --- |
|  | 14 (2.3%) | 64 (10.7%) | 521 (87%) | **All** |
| 0.87 | 1 (1.5%)  11 (2.5%) | 6 (9.1%)  36 (8.2%) | 59 (89.4%)  390 (89.3%) | **Age of menarche**  **<12 (n=69)**  **≥12 (n=448)** |
| 0.41 | 11 (2.1%)  3 (4.8%) | 52 (9.9%)  6 (9.7%) | 461 (88%)  53 (85.5%) | **Nulliparity**  **No (n=543)**  **Yes (n=62)** |
| 0.16 | 2 (5.75)  12 (2.2%) | 1 (2.9%)  57 (10.3%) | 32 (91.4%)  482 (87.5%) | **Multiparity (≥5 deliveries)**  **Yes (n=36)**  **No (n=569)** |
| 0.88 | 2 (3.6%)  7 (2.5%) | 4 (7.1%)  23 (8.3%) | 50 (89.3%)  249 (89.2%) | **Age at first delivery**  **≥30 (n=58)**  **<30 (n=284)** |
| 0.001 | 7 (1.6%)  6 (4.7 %) | 56 (12.5%)  4 (3.2%) | 385 (85.9%)  117 (92.1%) | **Menopause**  **Yes (n=464)**  **No (n=131)** |
| 0.30 | 5 (1.4%)  2 (3.2%) | 37 (10.8%)  10 (15.9%) | 302 (87.8%)  51 (80.9%) | **Early menopause (age<45)**  **No (n=353)**  **Yes (n=65)** |
| 0.36 | 6 (1.5%)  1 (6.3%) | 45 (11.5%)  2 (12.5%) | 340 (87%)  13 (81.2%) | **Late menopause (age>55)**  **No (n=401)**  **Yes (n=17)** |

Data on the initial endocrine therapy were not available for: all patients- n=21, age of menarche- n=14, number of deliveries- n=19, age of first delivery- n=7, menopause- n=20, age of menopause- n=11.
